# Elevated Serum Cortisol Levels in Patients with Focal Epilepsy, Depression, and Comorbid Epilepsy and Depression

**DOI:** 10.3390/ijms231810414

**Published:** 2022-09-08

**Authors:** Tatyana A. Druzhkova, Alexander A. Yakovlev, Flora K. Rider, Mikhail S. Zinchuk, Alla B. Guekht, Natalia V. Gulyaeva

**Affiliations:** 1 Research and Clinical Center for Neuropsychiatry of Moscow Healthcare Department, 115419 Moscow, Russia; 2 Department of Functional Biochemistry of Nervous System, Institute of Higher Nervous Activity and Neurophysiology, Russian Academy of Sciences, 117485 Moscow, Russia; 3 Department of Neurology, Neurosurgery and Medical Genetics, Pirogov Russian National Research Medical University, 119049 Moscow, Russia

**Keywords:** epilepsy, depression, hypothalamic-pituitary-adrenal axis, cortisol, adrenocorticotropic hormone, tumor necrosis factor α, BDNF, CNTF, lymphocytes, eosinophils, monocytes, neutrophils

## Abstract

Background: The hypothalamic-pituitary-adrenal (HPA) axis, inflammatory processes and neurotrophic factor systems are involved in pathogenesis of both epilepsy and depressive disorders. The study aimed to explore these systems in patients with focal epilepsy (PWE, *n* = 76), epilepsy and comorbid depression (PWCED *n* = 48), and major depressive disorder (PWMDD, *n* = 62) compared with healthy controls (HC, *n* = 78). Methods: Parameters of the HPA axis, neurotrophic factors, and TNF-α were measured in blood serum along with the hemogram. Results: Serum cortisol level was augmented in PWE, PWCED, and PWMDD compared with HC and was higher in PWMDD than in PWE. Serum cortisol negatively correlated with Mini–Mental State Examination (MMSE) score in PWE, and positively with depression inventory–II (BDI-II) score in PWMDD. Only PWMDD demonstrated elevated plasma ACTH. Serum TNF-α, lymphocytes, and eosinophils were augmented in PWMDD; monocytes elevated in PWE and PWCED, while neutrophils were reduced in PWE and PWMDD. Serum BDNF was decreased in PWE and PWCED, CNTF was elevated in all groups of patients. In PWE, none of above indices depended on epilepsy etiology. Conclusions: The results confirm the involvement of HPA axis and inflammatory processes in pathogenesis of epilepsy and depression and provide new insights in mechanisms of epilepsy and depression comorbidity.

## 1. Introduction

Epilepsy is one of most widespread neurological diseases; about 70 million people worldwide suffer from epilepsy. A heterogeneous disease, epilepsy may be associated with a variety of etiological factors (including genetic, infectious, traumatic, vascular, toxic, tumor, and many more causes). The current understanding of epilepsy is not limited by considering it just as a disease accompanied by seizures, but it also implies that epilepsy is frequently associated with comorbid cognitive and mental disorders [1]. The occurrence of comorbid mental illness in patients with epilepsy (PWE) is rather high: one in three patients has experienced a mental disorder during the lifetime, usually mood disorders and anxiety [2]. Temporal lobe epilepsy among all epilepsies is most frequently associated with psychiatric comorbidity, including depression and anxiety as well as with cognitive impairment [3,4]. Mental comorbid diseases affect the quality of life of PWE as well as the course of epilepsy. They are associated with the poorer tolerability of antiepileptic drug therapy, an increased risk of mortality, and a higher economic burden on both the patient/family and society [3,5].

PWE are at greater risk of developing mental disorders, and patients with primary mental disorders are at higher risk of developing epilepsy [6]. According to the bidirectional hypothesis, the close relationship between epilepsy and psychiatric features should consider common pathophysiology [3]. Indeed, epilepsy and mental diseases have multifaceted connections underlying these bidirectional relationships. Depression is one of most disabling comorbidities of epilepsy, and this relationship can only be explained by the occurrence of common multi-level mechanisms underlying the development of both diseases. Both clinical and experimental data confirm the existence of common pathogenetic mechanisms operating in both depressive and, most likely, anxiety disorders and epilepsy. There is a growing array of data demonstrating biochemical, neuropathological, and neurophysiological changes common to epilepsy and depression, substantiated by results obtained in animal models (see [7] for review). This may explain relatively high comorbidity of epilepsy and depression, their bidirectional relationship, and the detrimental effects of a previous history of depression on the course of convulsive disorder [4,6].

Comorbid temporal lobe epilepsy and depression are associated with dysfunction of the hypothalamic-pituitary-adrenocortical (HPA) axis [7]. Excessive glucocorticoids disrupt the function and impair the structure of the hippocampus, a brain region key to learning, memory, and emotions [8,9]. Selective vulnerability of the hippocampus to stress, mediated by the reception of glucocorticoid hormones secreted during stress, is the price of the high functional plasticity and pleiotropy of this limbic structure. Common molecular and cellular mechanisms include the dysfunction of glucocorticoid receptors, neurotransmitters, and neurotrophic factors, development of neuroinflammation, leading to neurodegeneration and loss of hippocampal neurons, as well as disturbances in neurogenesis in the subgranular neurogenic niche and formation of aberrant neural networks [10]. These glucocorticoid-dependent processes underlie altered stress response and the development of chronic stress-induced comorbid pathologies, in particular, temporal lobe epilepsy and depressive disorders.

The excessive secretion of stress hormones glucocorticoids (in particular, cortisol, the principal glucocorticoid in humans), released into the blood as a result of the HPA axis functioning has a continuous effect on the brain. The HPA dysfunction is associated with disturbances in both central and peripheral immune and inflammatory processes, and neurotrophic factors [11]. Deregulations of these systems reported by different groups have been revealed in both PWE and animal epilepsy models [12,13,14,15] as well as in patients with major depression disease (PWMDD) [16,17,18].

The aim of this study was to further explore the mechanisms of epilepsy and depression comorbidity in a comparative investigation of selected HPA axis indices, markers of immune system, and inflammatory and neurotrophic factors in PWE, PWMDD, and epilepsy with comorbid depression (PWCED), as compared with healthy controls (HC).

## 2. Results

### 2.1. Characteristics of the Patients and the Control Group

The demographic, clinical, biochemical data describing subjects of PWE, PWMDD, and PWCED, and the HC group, as well as information about treatment of the patients, are shown in Table 1. The patients in the groups studied did not significantly differ in age, BMI, gender, and education level.

Basic biochemical parameters (total bilirubin, glucose, creatinine, urea, cholesterol, triglycerides) as well as some hemogram indices (platelets, erythrocytes, hemoglobin, white blood cell count) did not differ between HC and patient groups.

Table 2 shows the nosological structure of the PWE group. Most blood serum or hemogram indices were similar in the three subgroups of PWE.

### 2.2. HPA Axis Indices

**Figure 1:**
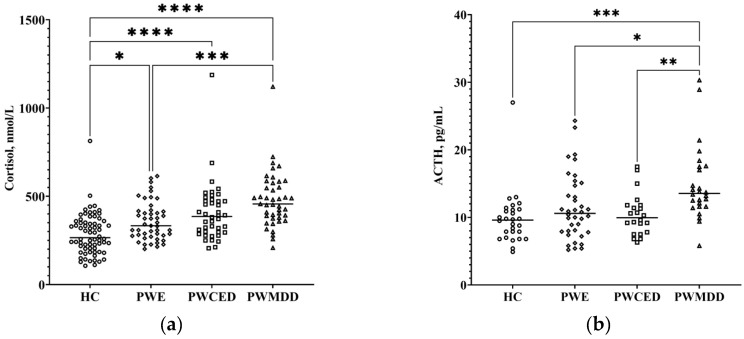
HPA axis indices in blood of PWE, PWECD, and PWMDD, and the HC group: (**a**) cortisol (serum); (**b**) ACTH (plasma). Differences between groups were assessed by one-way ANOVA and in case of significance, a Tukey post-hoc test was used. * *p* < 0.05, ** *p* < 0.01, *** *p* < 0.001, **** *p* < 0.0001.

Concentrations of cortisol in blood serum of PWE, PWECD, and PWMDD were augmented as compared with the HC group (Figure 1a; Table 1). When groups of patients were compared, in PWMDD serum, cortisol concentrations were significantly higher than in PWE. Though PWE, PWECD, and PWMDD included patients with rather high ACTH levels, only in PWMDD blood plasma ACTH was significantly higher than in HC and both groups with epilepsy (Figure 1b; Table 1). The cortisol/ACTH ratio was similar in all groups. HPA indices did not depend on the etiology of epilepsy (Table 2).

### 2.3. Neurotrophic Factors

**Figure 2:**
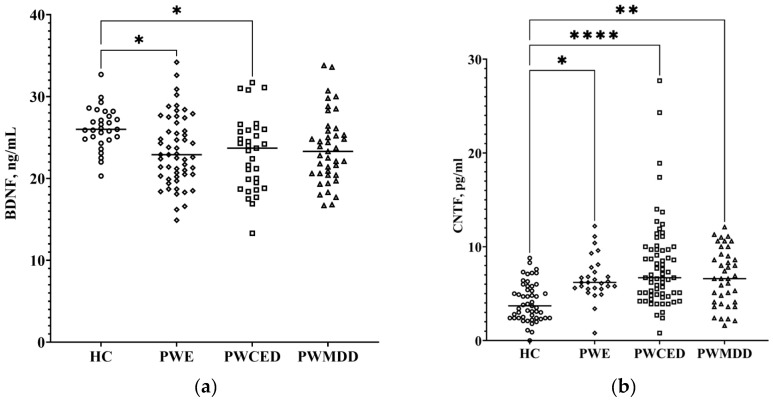
Neurotrophic factors in blood serum of PWE, PWECD, PWMDD, and the HC group: (**a**) BDNF; (**b**) CNTF. Differences between groups were assessed by one-way ANOVA and in case of significance, a Tukey post-hoc test was used. * *p* < 0.05, ** *p* < 0.01, **** *p* < 0.0001.

Blood serum BDNF was significantly lower in patients with epilepsy (PWE, PWECD) (Figure 2a; Table 1), while in the PWMDD group, the decrease showed a statistically significant trend (*p* = 0.055). CNTF levels were higher in PWE, PWECD, and PWcDE as compared with the HC group (Figure 2b; Table 1). GDNF and NGF levels in blood of patients and HC did not differ (Table 1). BDNF and CNTF levels did not depend on the etiology of epilepsy, while GDNF level was higher in two PWE subgroups as compared with the “trauma” subgroup (Table 2).

### 2.4. TNF-α

**Figure 3:**
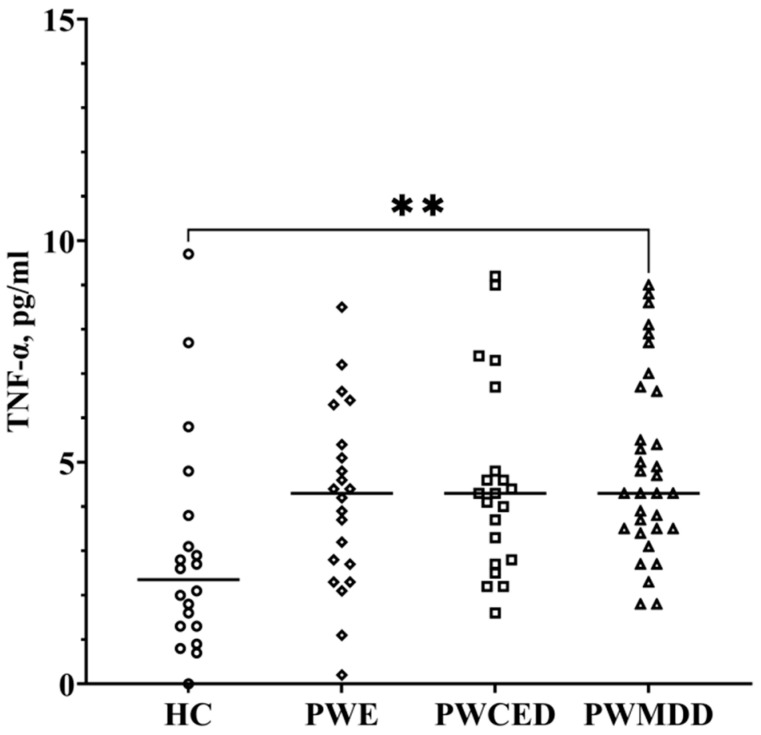
TNF-α in blood serum of PWE, PWECD, and PWMDD, and the HC group. Differences between groups were assessed by one-way ANOVA and in case of significance, a Tukey post-hoc test was used. ** *p* < 0.01.

Though the dispersion of TNF-α concentrations in blood serum of the HC group and in the patient groups was large, a statistically significant increase was revealed in PWMDD as compared with the HC group (Figure 3; Table 1). TNF-α levels did not depend on the etiology of epilepsy (Table 2).

### 2.5. Hemogram

**Figure 4:**
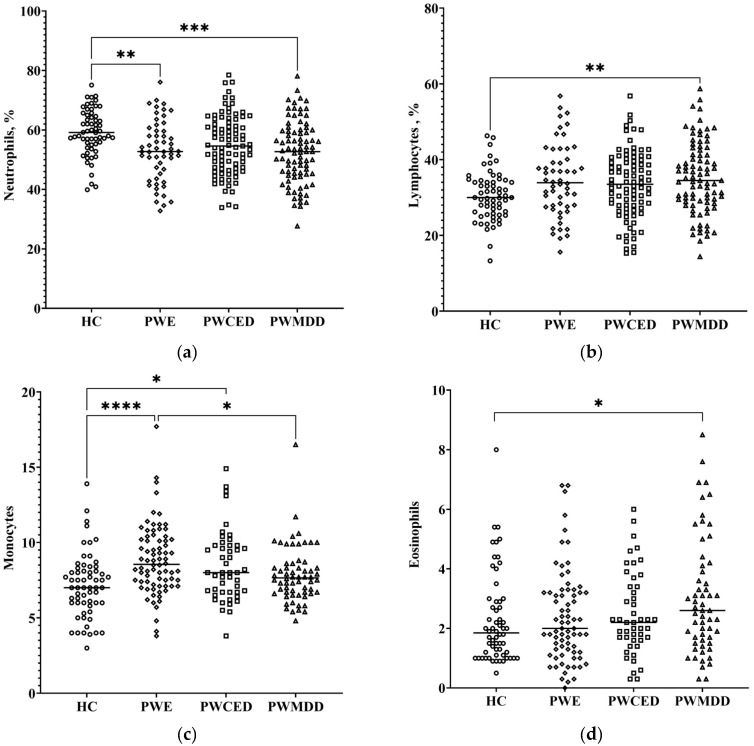
HPA axis in blood serum of PWE, PWECD, and PWMDD: (**a**) neutrophils; (**b**) lymphocytes; (**c**) monocytes; (**d**) eosinophils. Differences between groups were assessed by one-way ANOVA and in case of significance, a Tukey post-hoc test was used. * *p* < 0.05, ** *p* < 0.01, *** *p* < 0.001, **** *p* < 0.0001.

The hemogram showed significant changes in groups of patients compared with the HC group (Figure 4a–d, Table 1). Neutrophils were reduced in PWE and PWMDD, while the trend to decrease in PWCED group did not reach significance (Figure 4a). PWMDD showed augmented lymphocytes (%), and eosinophils were augmented in (Figure 4b,d). Monocytes were elevated in PWE and PWCED (Figure 4c). Though monocytes in PWMDD did not differ from HC, this index was higher as compared with PWE. Basophil count did not change in either group of patients. Most hemogram parameters did not depend on the etiology of epilepsy, but platelet count was lower in the two PWE subgroups as compared with the “trauma” subgroup (Table 2).

### 2.6. Correlations

In PWE, cortisol negatively correlated with Mini–Mental State Examination (MMSE) score (r = −0.457, *p* = 0.007), while in PWMDD, cortisol positively correlated with Beck depression inventory—II (BDI-II) 2 score (r = 0.328, *p* = 0.028). In all subjects under study, cortisol level positively correlated with ACTH (r = 0.585, *p* = 0.000), and this correlation was evident in all groups (HC, r = 0.549, *p* = 0.002; PWE, r = 0.526, *p* = 0.004; PWECD, *p* = 0.710, *p* = 0.000; PWMDD, *p* = 0.460, *p* = 0.009).

## 3. Discussion

### 3.1. HPA Axis Dysfunction in Depression and Epilepsy

The body’s physiological response to any significant influence (stress) is mediated by the HPA axis and involves a hormonal cascade, including corticotropin releasing hormone (CRH), adrenocorticotropin releasing hormone (ACTH), and cortisol. The HPA axis is involved in the pathophysiology of many neuropsychiatric disorders. Increased HPA axis activity is observed during chronic stress, which plays a key role in the pathophysiology of both depression and epilepsy, leading to cognitive dysfunction and reduced mood [7].

A high degree of dysregulation of the HPA axis is believed to be one of the most consistent findings in the biology of depression [19]. Altered activity of the HPA axis is a commonly observed neuroendocrine abnormality in patients suffering from major depression (MDD), and changed cortisol secretion was found in as many as 80% of depressed patients [20]. Both centrally released CRH and increased levels of cortisol contribute to the signs and symptoms of depression. Cortisol is regarded as a biological marker that can help assessing the risk of mental illness, its future onset, and the severity of symptoms [21,22]. Interestingly, the more marked depressed patients’ alterations in the HPA axis, the less likely they are to profit from antidepressants [23]. In our study, PWMDD demonstrated augmented blood cortisol and ACTH, confirming many previous reports. Correlation of cortisol with BDI-II score in PWMDD confirms that the HPA axis dysfunction is intimately involved in depression symptoms in MDD.

The involvement of the HPA axis dysregulation in the development of MDD has been recognized for a long time, while studies on the association of HPA axis functioning and epilepsy started quite recently. Stress is a known precipitating factor for seizures in individuals suffering from epilepsy. Severe acute stress and persistent exposure to stress may increase susceptibility to seizures, thereby resulting in a higher frequency of seizures. Cano-López and González-Bono [24] have analyzed data of 38 studies on cortisol levels and seizures in adults with epilepsy. Higher basal cortisol levels were found in PWE as compared with respective HC groups in studies with the most homogeneous samples (45% of 38 total studies), while seizures were related to increases in cortisol levels in 77% of studies. In studies using self-reported stress, high seizure frequency was related to increased blood cortisol and low functional brain connectivity. The authors consider epilepsy a chronic stress model [24] and we fully agree with this [7]. In the present study, we confirmed augmented levels or cortisol in blood serum of PWE.

Chronic stress, a recognized major player in epileptogenesis and epilepsy, can also impair cognitive function [25]. Increased cortisol levels have been reported in epilepsy patients, and animal models evidence that aggravating effects of stress on memory and synaptic plasticity are mediated via glucocorticoids [7]. A direct negative glucocorticoid effect on synaptic potentiation in the human cortex and chronic activation of glucocorticoid receptors in patients with temporal lobe epilepsy has been demonstrated [26]. In patients with drug-resistant epilepsy, memory performance was negatively related to the cortisol AUC and trait anxiety, both being reliable predictors of memory performance, especially in patients with left-hemisphere focus [27]. Correlation of cortisol with MMSE score in PWE found in the present study confirm that HPA axis dysfunction may be associated with cognitive impairments in epilepsy.

The protracted exposure to cortisol may not only exacerbate epilepsy, but may also increase the predisposition to mood disorders. The temporal lobe is intimately involved in both epilepsy and depression. Hyperactivity of the HPA axis negatively affects the structure and function of the temporal lobe of the brain, in particular, the hippocampus [28,29,30]. Seizures per se induce damage of temporal lobe structures and disinhibit the HPA axis, contributing to a vicious cycle of neuronal damage and increasing susceptibility for subsequent seizures and psychiatric comorbidity [31]. The pathways of stress and anxiety/depression involve the HPA axis, and epilepsy is a unique illness that is intimately intertwined with stress and anxiety/depression not only as a result of the disease process but also as a cause of disease exacerbation [32]. In other words, both depression and epilepsy are stress-related diseases sharing molecular mechanisms of stress response, and this is a general explanation for the comorbidity of epilepsy and depressive disorders [7]. Obviously, HPA axis dysfunction is characteristic for both PWMDD and PWE; however, higher cortisol in PWMDD as compared with PWE, together with augmented serum ACTH in PWMDD suggests not only different degrees but, most probably, distinctions in the mechanisms of HPA axis malfunction. Correlations of serum cortisol with MMSE score in PWE and with BDI-II score in PWMDD confirms associations of augmented cortisol with cognitive and emotional decline in epilepsy and depression, respectively.

### 3.2. Inflammatory and Immune Processes in Depression and Epilepsy

Low-grade (sterile) neuroinflammation has been suggested as an important mechanism underlying many psychiatric diseases as well as cognitive disorders. Mood disorders are associated with chronic low-grade systemic inflammation accompanied by increased circulating pro-inflammatory cytokines and other pro-inflammatory mediators (interleukins, TNF-α), targeting all tissues including the brain [33,34]. Pro-inflammatory cytokines regulate mood and cognition by influencing neurotransmitter levels, activating stress-responsive endocrine axes and promoting depression-like behavior. Altered leukocyte population frequencies in blood, accumulation of immune cells in the brain, and activation of these immune cells are among major events associated with systemic inflammation. Many patients with mood disorders exhibit immune-related abnormalities, including elevated levels of proinflammatory cytokines, monocytes, and neutrophils in the peripheral circulation as well as dysregulation of neuroglia and blood–brain barrier function [35].

Inflammation has been thought for a long time to contribute to MDD, a widespread, severe, and disabling neuropsychiatric disorder with a heterogenous etiology. Depression is associated with aberrant levels of inflammatory markers in the peripheral blood, cerebrospinal fluid (CSF), and postmortem brain of respective patients [36]. Multiple studies including meta-analyses have reported increased peripheral levels of acute phase proteins, and pro-inflammatory cytokines, particularly IL-1β, TNF-α, and IL-6, in major depression disease. A meta-analysis of mean differences and variability in 5166 patients and 5083 controls showed that TNFα was significantly higher in patients with depression, confirming depression as a pro-inflammatory state [37]. Our data confirm an augmented level of TNF-α in PWMDD.

Increases in IL-6 and TNF-α levels in CSF and brain parenchyma are considered markers of central inflammation in depression in the context of a possible increased microglia activity and reduction of astrocyte and oligodendrocyte markers [38,39]. Along with evidence of monocytic, lymphocytic, and microglial activation this suggests an activated inflammatory response system in MDD [40]. Both high levels of cortisol and proinflammatory cytokines are involved in the enhanced breakdown of tryptophan in patients with depression, thus affecting the kynurenine pathway [40]. Impaired immunoregulatory mechanisms are believed to induce systemic neuroinflammation affecting mechanisms of stress-response, and to cause microglial activation and decrease of trophic support.

Many studies have demonstrated critical involvement of stress-induced immune responses in depressive- and anxiety-like behaviors. Sympathetic activation and glucocorticoids evoke the mobilization of peripheral neutrophils and monocytes to the circulation. These myeloid cells may promote depressive- and anxiety-like behaviors by infiltrating the brain’s perivascular space, releasing cytokines, and affecting vascular endothelial functions [41]. Stress activates brain microglia via innate immune receptors TLR2/4, and the activated microglia promote depressive-like behavior by inducing a number of region-specific neuroinflammatory processes. These rodent findings on peripheral and brain inflammation are believed to be translatable to human depression.

Immunity and inflammation appear to be an integral part of the pathogenic processes associated with seizures, particularly with refractory epilepsy. The contribution of the innate and adaptive immune system to ictogenesis and epileptogenesis is vigorously studied. There is enough evidence on the activation of these branches of the immune system in the epileptic brain, the fundamental role of neuroinflammation in the development of epilepsy being actively discussed [42,43]. Neutrophils (most abundant leucocytes in human circulation and the first line of defense against microbial challenge) and TNFα are regarded as central regulators of neuronal hyperexcitability of diverse etiology [44]. Other immune cells can impact neurological conditions too, and peripheral immune cell invasion into the brain is implicitly involved in epilepsy. In particular, peripheral monocytes (responsible for innate immunity), which can be recruited to the central nervous system, infiltrate into brain parenchyma after seizures and function together with their “immunological cousins”, i.e., microglia [45,46]. Epileptic seizures induce brain injury, in particular, stimulating neuroimmune response with activation of microglia and astrocytes producing and releasing inflammatory mediators. A wide spectrum of neuroinflammatory pathways is involved in seizures recurrence and neurodegeneration associated with epilepsy, the neuroimmune response being commonly observed in the epileptic brain [47].

Preclinical studies suggest that neuroinflammation arising in brain areas of seizure onset and generalization is a key contributor to neuronal hyperexcitability underlying seizure generation [48]. Pre-clinical and clinical data show a positive feedback cycle between neuroinflammation and epileptogenesis. Prolonged or recurrent seizures and brain injuries lead to upregulation of proinflammatory cytokines and activated immune responses to further increase seizure susceptibility, promote neuronal excitability, and induce blood–brain barrier breakdown [49]. Epileptic seizures are associated with elevated levels of proinflammatory cytokines, including TNF-α, which mediate the impact of neuroinflammation hyperexcitability of the brain and epileptogenesis [50]. TNF-α is among the most extensively investigated proteins in the studies on inflammatory mediators in human epilepsy [36,51]. TNF-α expression was shown to be important in posttraumatic epileptogenesis [52]. Though the mean TNF-α level in our study was 1.45 times higher than in the HC group, this difference did not reach statistical significance, while a 1.82-fold increase in PWCED was significant as compared with the HC group. Güneş and Büyükgöl [53] reported a relationship between generalized epileptic seizure and significant inflammation biomarkers: neutrophil/lymphocyte ratio, platelet/lymphocyte ratio, and neutrophil mediated inflammation. However, we were unable to confirm these data in PWE with focal seizures. A reduction of neutrophils revealed in our study may reflect a response to chronic stress load in PWE and PWCED. Augmentation of monocytes, which are also part of the innate immune response and function to regulate cellular homeostasis, especially in the setting of inflammation, substantiates challenges to immune system in epilepsia (PWE and PWCED groups).

Alvim et al. [54] investigated the association between plasma inflammatory and neurotrophic markers, seizure frequency, and chronic epilepsy subtypes in a large cohort of PWE. They showed that plasma levels of BDNF, NT3, NGF, and sTNFr2 were higher, whereas IL-2, IL-4, IL-6, IL-10, IL-17, IFNγ, TNFα, CNTF, and sTNFr1 were lower in PWE than controls. These data contradict the results of many studies, including the present study and our previous results, which showed an increase of CNTF level in blood serum of PWE [55]. There may be many reasons for these discrepancies related to the specific features of the PWE cohort studied. However, importantly, similarly to our data, changes in plasma inflammatory and neurotrophic markers, whatever they were, did not depend on the etiology of epilepsy [56], supporting our results that the revealed changes were related to epilepsy in general, independently of etiology.

### 3.3. Neurotrophic Factors in Depression and Epilepsy

Brain-derived neurotrophic factor (BDNF), a member of the neurotrophic factor family, plays an important role in the survival, growth, and differentiation of neurons. BDNF is a critical regulator of various types of neuronal plasticity in the brain. Several in vitro, ex vivo, and in vivo studies suggest an involvement of BDNF in the pathophysiology of epilepsy [56]. However, two controversial views (BDNF inhibits or promotes epileptogenesis) still exist [57]. This discrepancy is most probably related to different role of BDNF in specific types of epilepsy and epileptogenesis and different expression and functions of BDNF in specific brain regions during the time course of epilepsy development [7]. Meta-analysis shows that, usually, PWE had BDNF levels similar to general population, although patients with partial epilepsy showed lower BDNF levels [58]. In our study, serum BDNF levels in PWE did not differ from that in the HC group.

Neurotrophic factors, particularly BDNF, have been associated with depression and antidepressant drug action. Multiple preclinical and clinical studies have implicated impaired BDNF signaling through its receptor TrkB in the pathogenesis of depression, while increased expression and signaling of BDNF has been implicated in antidepressant-related events, and plasticity has increasingly been connected with antidepressant action [59]. The results of many clinical and animal studies confirm that BDNF and inflammation are two important risk factors in the pathogenesis of depression. Indeed, BDNF has both pro- and anti-depressant effects, dependent on brain region, confirming a strong, though region-specific, contribution of BDNF to depression pathogenesis [60]. Elevated levels of inflammatory mediators reduce expression of BDNF, while BDNF may play a negative regulatory role in neuroinflammation [61]. BDNF and cortisol are believed to be biomarkers of MDD [34,62], in particular, serum cortisol and mature BDNF are among the specific biomarkers with a potential on the evaluation of MDD chronicity [63]. Current research has identified associations between changes in serum BDNF levels and post-stroke depression [64]. In our study, serum BDNF was reduces in both groups with epilepsy, PWE, and PWCED; in the PWMDD group, the decrease showed a statistically significant trend, the mean value being similar to that of two groups with epilepsy. In general, our results confirm the results of many studies, including those cited above.

As noted above, neurotrophic factors may influence affective behavior including depression and anxiety. CNTF plays an important role in the regulation of neuronal development, neuroprotection, and may also influence cognitive processes. Using mice deficient in CNTF (CNTF −/− mice), Peruga et al. [65] have demonstrated that CNTF plays an essential role in the maintenance of hippocampal functions, thus modulating affective behavior in rodent models of anxiety and depression. CNTF is produced by astrocytes and promotes neurogenesis and neuroprotection. CNTF was shown to be a key sex-specific regulator of depressive-like behavior in mice controlling CNTF-mediated mechanism in stress-induced depressive-like behavior [66]. Earlier, we have shown that patients with MDD manifest an elevated basal HPA axis activity and CNTF levels, while mild stress induces increase in TNF-α in these patients but not in the HC group [67]. Recently, Shpak et al. [55] demonstrated an almost two-fold increase of CNTF in blood serum of PWE, suggesting that high CNTF in blood serum and lacrimal fluid may be a biomarker of focal epilepsy. No association of CNTF levels with age, gender, or clinical parameters, as well as depression occurrence, was found. We confirmed these data in the present study showing augmented levels of CNTF, a pluripotent neurotrophic factor and a pleiotropic cytokine of the interleukin-6 family, in PWE, PWCED, and PWMDD. Unfortunately, there is not enough available information to explain this phenomenon, since we have no data on the function of blood CNTF. CNTF may function as neuroprotector; however, it belongs to the IL-6 family of cytokines which may promote chronic diseases [68].

In this study, we additionally analyzed subgroups of patients with different etiology of epilepsy within the PWE group. Three subgroups were selected, “trauma” (traumatic brain injury and cerebrovascular disorders, including stroke), “tumors”, and PWE with not established etiology or multiple reasons. Though most parameters measured were not different between these three subgroups, compared to the “trauma” group, two others had a lower GDNF level and the subgroup with not established etiology displayed lower TSH3 level and higher platelet count. As compared with the “tumor” subgroup, two others had lower triglycerides. There are no available data to reasonably discuss this phenomenon; however, they are established in our study, and should be reproduced or not confirmed in other cohorts of PWE to be correctly interpreted.

## 4. Materials and Methods

### 4.1. Subjects

A group of 186 patients over 18 years old diagnosed with focal epilepsy (PWE, n = 76), with major depressive disorder (PWMDD, n = 62) and with focal epilepsy and comorbid (PWCED, n = 48), was recruited at the Moscow Research and Clinical Center for Neuropsychiatry between November 2019 and June 2021.

Inclusion criteria for the group with epilepsy were: focal epilepsy, thoroughly diagnosed through consensus by at least two experienced neurologists according to the criteria for epilepsy, as based upon the International League Against Epilepsy (ILAE) classification [69,70]. All PWE underwent electroencephalography (EEG) and magnetic resonance imaging (MRI) of the brain. Basic demographic data and clinical parameters of epilepsy were assessed (Table 2). Subjects were excluded from the study if they had no records of seizure frequency, generalized, combined or unknown epilepsy, significant psychiatric comorbidity (excluding depression), history of psychogenic nonepileptic seizures, presence of serious somatic, or neurological or systemic disorders.

All patients were examined by an experienced psychiatrist to diagnose depression and exclude other psychiatric comorbidities. Inclusion criteria for the group with MDD were: (1) the diagnosis of current depressive episode (MDD), (2) age 18 years and above, (3) fluency in the Russian language, and (4) the ability to provide an informed consent and comply with the study protocol. The exclusion criteria were (1) cognitive impairment (score less than or equal to 24 on the Mini-Mental State Examination (MMSE), (2) current or past psychotic disorders, alcohol and substance use disorders, or manic/hypomanic symptoms/episodes, and (3) severe concomitant somatic (e.g., diabetes mellitus, autoimmune or oncological diseases) and neurological (e.g., Alzheimer’s and Parkinson’s diseases) disorders. Persons with initial or mild manifestations of somatic diseases, such as essential hypertension, ischemic heart disease, or cardiac arrhythmias, were not excluded.

A mental disorder diagnosis was established by a psychiatrist using a Mini-International Neuropsychiatric Interview (MINI v 7.0.2). MINI is a widely used structured diagnostic interview developed for the assessment of the most common mental disorders. MMSE is a widely used 30-point test of cognitive function, which evaluates orientation, attention, memory, language, and visual-spatial skills [71]. We used a single cutoff < 24 to detect those with cognitive impairment.

The patients were not treatment naïve and received appropriate medications (treatment as usual) prescribed by an experienced psychiatrist. The Russian version of the Beck depression inventory—II (BDI-II) was used to evaluate the severity of depression [72]. The BDI-II is a widely used 21-item multiple-choice self-report inventory measuring the severity of depression in adults. All patients signed an informed consent form prior to participating in the study.

Seventy-eight practically healthy people of similar age and sex with no signs of mental disorder, both at present and in their medical history, formed a control group.

This study adhered to the tenets of the Declaration of Helsinki and had local ethics committee approval (#42, 19.08.2019) with informed consent obtained from all subjects.

### 4.2. Assessment of Biochemical Indices and Hormones

Biochemical and hormonal parameters were measured in blood serum obtained from fasting morning venous blood. Samples were collected in Gel/Clotting activator S-Monovette tubes and centrifuged at 2000× *g* for 10 min at 8 °C on an Allegra X-30R Centrifuge (Beckman Coulter, Brea, CA, United States).

Biochemical parameters (glucose, bilirubin, creatinine, triglycerides, cholesterol, urea, ALT, AST, GGT, and ALP) were determined in blood serum on a biochemical automated analyzer Beckman Coulter AU 680 (Beckman Coulter, Brea, CA, USA) using corresponding kits (Beckman Coulter, United States). Cortisol, thyroid stimulating hormone (TSH), and free thyroxine (FT4) were measured in blood serum via competitive enzyme immunoassay using applicable kits (Beckman Coulter, United States) and an ACCESS^®^ 2 immunoassay system (Beckman Coulter, United States). The concentrations of ciliary neurotrophic factor (CNTF), brain derived neurotrophic factor (BDNF) were determined by enzyme-linked immunosorbent assay (ELISA) in blood serum using corresponding Quantikine ELISA test systems (R&D Systems, Minneapolis, MN, United States); the concentration of tumor necrosis factorα (TNF-α) was determined with corresponding Human high sensitivity ELISA kits (eBioscience, Bender MedSystems GmbH, Vienna, Austria); adrenocorticotropic hormone (ACTH) was assessed in EDTA plasma samples using enzyme immunoassay kits from Biomerica (Irvine, CA, USA). CNTF, BDNF, TNF-α, and ACTH parameters were measured on an automated enzyme immunoassay analyzer (ChemWell 2910, Awareness Technologies Inc., Palm City, FL, USA).

Complete blood count with differential white blood cell count (CBC with diff) and hemogram were performed on an automated analyzer LH-500 (Beckman Coulter, United States).

### 4.3. Statistical Analysis

Statistical analysis was performed in STATISTICA for Windows ver. 12.5 (StatSoft Inc., Tulsa, OK, USA) and GraphPad Prism ver. 8 (GraphPad Software, USA). Results are presented as mean ± standard deviation (M ± SD). The statistical significance of differences between groups was determined using one-way analysis of variance (ANOVA). Multiple comparisons were made using a Tukey test. To reveal the relationship between the variables, we performed the correlation analysis with the calculation of the Spearman rank correlation coefficient (r). Outliers were determined in GraphPad Prism using the built-in ROUT algorithm (Q = 1%). Differences were considered significant at a significance level of *p* < 0.05.

## 5. Conclusions

Depression, a multicausal disorder, is associated with risks to develop epilepsy, stroke, dementia, diabetes, cancer, and other diseases [73]. As an endocrinological and stress-related disorder, depression is connected with HPA axis hormones, as well as many others. As an inflammatory disorder, it is mediated by pro-inflammatory mediators including interleukins and TNF-alpha. The neurodegenerative hypothesis of depression explains decreased hippocampal volumes and impairments of neurotrophic support by BDNF. Epilepsy, a stress-related disorder as well, shares major links of MDD pathogenesis, HPA axis disturbances, and inflammatory/immune alterations [7]. It appears that changes in these systems in PWE and PWMDD include both identical processes, important for comorbidity, and specific for either epilepsy or depression. Similarly to PWMDD, PWE and PWECD have augmented cortisol levels, though HPA axis alterations seem to be deeper in PWMDD. Correlations of cortisol with MMSE score in PWE and BDI-II score in PWMDD suggest that the HPA axis dysfunction is not only intimately involved in depression symptoms in MDD, but also may be associated with cognitive impairments in epilepsy. PWE, PWMDD, and/or PWCED as well share similar tendency to increase of monocytes and serum CNTF along with decrease of blood neutrophyls; however, pro-inflammatory trends are more expressed in PWMDD as compared with PWE. The independence of the HPA axis indices, inflammatory, immune parameters, and neurotrophic factors (BDNF, CNTF, and NGF) in PWE on the etiology of epilepsy suggests that the alterations observed may result rather from the disease burden, including stress load and seizures, than from the specific pathogenetic mechanisms underlying definite forms of focal epilepsy.

## Figures and Tables

**Table 1:** Patient and control group characteristics.

Parameter/ Group	HC *n* = 78	PWE *n* = 76	PWCED *n* = 48	PWMDD *n* = 62
**demographic data**				
Age, years	44.3 ± 13.4	42.2 ± 13.7	48.4 ± 12.5	44.6 ± 11.3
BMI	25.5 ± 4.6	26.3 ± 4.2	25.6 ± 5.00	24.1 ± 5.9
gender (male/female), %	37/63	50/50	25/75	30/70
Education(sec./higher), %	58/42	55/45	60/40	44/56
Employment (−/+), %	6/94	66/34	50/50	63/37
**clinical data**				
take antipsychotics, %		30	71	84
take antidepressants, %		75	92	98
take tranquilizers, %		37	52	55
take anti-seizure medication, %		93	94	13
disease duration		15.4 ± 12.9	14.9 ± 12.4	8.14 ± 5.6
BDI-II		8.7 ± 6.1	25.8 ± 10.3 ***	32.3 ± 8.00 **
MMSE		27.6 ± 2.3	26.7 ± 2.3 ***	28.5 ± 1.4
**blood serum (plasma) indices**				
TSH3, ulU/mL	2.1 ± 1.2	2.1 ± 1.1	2.3 ± 2.2	2.5 ± 1.9
ACTH, pg/mL	9.8 ± 4.0	11.6 ± 4.9 ***	10.4 ± 3.1 ***	13.8 ± 5.8 *
cortisol, mmol/L	288.3 ± 115.6	386.3 ± 135.2 *	433.3 ± 174.3 *	495.7 ± 142.5 *^;^**
BDNF, ng/mL	26.1 ± 2.6	23.4 ± 4.3 *	23.1 ± 4.5 *	23.7 ± 4.3
CNTF, pg/mL	4.1 ± 2.1	7.9 ± 2.6 *	8.3 ± 5.1 *	6.4 ± 2.8 *
GDNF, pg/mL	169.5 ± 53.1	143.9 ± 44.6	158.4 ± 53.9	152.8 ± 37.9
NGF, pg/mL	29.5 ± 10.1	27.7 ± 12.2	24.2 ± 7.3	24.4 ± 8.3
TNF-α, pg/mL	2.9 ± 2.4	4.2 ± 2.0	4.5 ± 1.93	5.1 ± 2.8 *
total bilirubin, µmol/L	12.7 ± 3.7	12.3 ± 5.1	10.8 ± 4.7	13.9 ± 6.7
glucose, mmol/L	5.1 ± 0.6	5.5 ± 1.0	5.4 ± 0.6	5.4 ± 1.0
creatinine, µmol/L	85.1 ± 13.1	84.4 ± 13.8	80.5 ± 11.3	83.2 ± 13.5
urea, mmol/L	5.2 ± 1.0	4.2 ± 1.4	4.0 ± 1.2	3.9 ± 1.1
cholesterol, mmol/L	5.1 ± 0.7	6.0 ± 1.3	6.1 ± 1.6	5.4 ± 1.2
triglycerides, mmol/L	1.3 ± 0.54	1.4 ± 1.0	1.4 ± 1.3	1.3 ± 0.9
**hemogram**				
platelets, PLT, 10^3^/µL	237.8 ± 54.1	226.4 ± 63.9	244.2 ± 62.5	243.0 ± 59.2
erythrocytes, RBC, 10^6^/µL	4.7 ± 0.5	4.7 ± 0.5	4.5 ± 0.4	4.7 ± 0.6
hemoglobin, Hb, g/L	141.3 ± 12.8	143.3 ± 14.9	135.5 ± 12.0	141.9 ± 14.2
while blood cell, WBC, 10^3^/µL	6.4 ± 1.7	5.8 ± 1.7	6.0 ± 1.9	6.4 ± 1.6
neutrophils, NE, %	59.2 ± 7.7	54.0 ± 10.3 *	54.6 ± 10.3	53.0 ± 10.9 *
lymphocytes, LY, %	30.4 ± 6.4	33.8 ± 9.6	33.6 ± 8.3	35.4 ± 9.9 *
monocytes, MO, %	7.2 ± 2.1	8.8 ± 2.3 *	8.5 ± 2.32 *	7.9 ± 1.9
eosinophils, EO, %	2.3 ± 1.5	2.4 ± 1.6	2.5 ± 1.4	3.1 ± 2.0 *
basophils, BA, %	0.8 ± 0.6	0.9 ± 0.51	0.9 ± 0.5	0.9 ± 0.5
PLT/NE, PNR	66.9 ± 21.1	80.1 ± 37.4 *	83.2 ± 32.5 *	79.1 ± 32.1
PLT/LY, PLR	131.2 ± 39.1	132.0 ± 65.0	135.9 ± 53.3	118.4 ± 42.7
PLT/MO, PMR	36.4 ± 13.9	27.5 ± 11.2 *	31.1 ± 11.8	32.5 ± 11.0

The data are presented as mean ± SD.* *p* < 0.005 compared to HC; ** *p* < 0.005 compared to PWE; *** *p* < 0.005 compared to PWE and PWMDD. Differences between groups were assessed by one-way ANOVA and in case of significance, a Tukey post-hoc test was used. BDI-II—depression inventory–II, TSH3—thyroid-stimulating hormone, ACTH—adrenocorticotropic hormone, BDNF—brain-derived neurotrophic factor, CNTF—ciliary neurotrophic factor, GDNF—glial-derived neurotrophic factor, NGF—nerve growth factor, TNF-α—tumor necrosis factor-α.

**Table 2:** Characteristics of patients with focal epilepsies.

Parameter/ Group	Etiology of Epilepsy		
	Traumatic Brain Injury, Cerebrovascular Disorders, Including Stroke *n* = 38	Tumors *n* = 20	Reason Not Established or Multiple *n* = 66
**Demographic data**			
Age, years	45.4 ± 11.5	46.9 ± 14.9	42.7 ± 14.1
BMI	25.8 ± 4.5	25.5 ± 3.3	26.4 ± 4.9
gender (male/female), %	55/45	25/75	36/64
Education (secondary/higher), %	63/37	55/45	54/46
Employment (−/+), %	55/45	55/45	64/36
**Clinical data**			
take antipsychotics, %	47	50	44
take antidepressants, %	71	85	86
take tranquilizers, %	32	40	50
take anti-seizure medication, %	92	95	94
BDI-II	14.8 ± 9.6	20.8 ± 13.8	14.1 ± 11.4
MMSE	26.6 ± 2.9	27.1 ± 2.2	27.7 ± 2.2
disease duration	13.7 ± 10.8	13.3 ± 10.0	16.6 ± 13.1
**seizure type**			
focal onset aware seizure, %	34	25	33
focal onset impaired/awareness seizure, %	45	55	65
focal to bilateral tonic-clonic seizure, %	79	75	73
**seizure frequency**			
absence in the last year, %	11	20	18
less than one per year, %	50	50	32
1–3 per month, %	21	5	15
more than one per week, %	8	15	23
**blood serum (plasma) indices**			
TSH3, ulU/mL	2.7 ± 2.2	2.0 ± 1.2	1.9 ± 1.1 *
ACTH, pg/mL	12.2 ± 3.8	9.9 ± 2.2	11.0 ± 4.6
cortisol, mmol/L	392.1 ± 116.4	384.9 ± 99.6	414.5 ± 182.3
BDNF, ng/mL	23.7 ± 4.4	22.5 ± 2.6	23.2 ± 4.8
CNTF, pg/mL	8.3 ± 4.7	7.2 ± 2.6	7.0 ± 3.0
GDNF, pg/mL	201.2 ± 53.1	123.4 ± 42.3 *	141.7 ± 40.8 *
NGF, pg/mL	26.3 ± 4.0	28.7 ± 13.7	26.4 ± 20.4
TNF-α, pg/mL	5.7 ± 2.1	5.0 ± 2.0	4.0 ± 1.8
total bilirubin, µmol/L	12.4 ± 4.3	12.2 ± 5.6	11.1 ± 5.2
glucose, mmol/L	5.7 ± 1.3	5.6 ± 0.7	5.3 ± 0.5
creatinine, µmol/L	83.2 ± 17.7	81.4 ± 11.3	83.3 ± 16.1
urea, mmol/L	4.0 ± 1.2	4.3 ± 1.5	4.1 ± 1.3
cholesterol, mmol/L	5.8 ± 1.4	6.6 ± 1.7	6.0 ± 1.3
triglycerides, mmol/L	1.2 ± 0.8 **	2.2 ± 1.9	1.1 ± 0.4 **
**hemogram**			
platelets, PLT, 10^3/^µL	211.5 ± 66.4	243.1 ± 69.5	242.9 ± 57.9 *
erythrocytes, RBC, 10^6/^µL	4.7 ± 0.4	4.6 ± 0.4	4.6 ± 0.5
hemoglobin, Hb, g/L	143.7 ± 12.8	137.2 ± 14.1	139.2 ± 15.0
while blood cell, WBC, 10^3/^µL	5.9 ± 1.7	5.4 ± 1.7	5.1 ± 1.8
neutrophils, NE, %	53.6 ± 10.7	53.9 ± 7.8	54.8 ± 10.8
lymphocytes, LY, %	34.4 ± 9.4	33.8 ± 7.2	33.4 ± 9.5
monocytes, MO, %	8.5 ± 2.5	8.8 ± 1.75	8.6 ± 2.36
eosinophils, BA, %	2.8 ± 1.6	2.5 ± 1.36	2.2 ± 1.43
basophils, BA, %	0.7 ± 0.3	0.8 ± 0.34	1.0 ± 0.60
PLT/NE, PNR	75.8 ± 40.0	89.4 ± 31.1	81.1 ± 33.9
PLT/LY, PLR	114.1 ± 40.6	143.8 ± 51.3	141.7 ± 70.1
PLT/MO, PMR	27.2 ± 12.2	29.0 ± 11.5	29.9 ± 11.1

The data are presented as mean ± SD.* *p* < 0.05 compared to the “trauma” group; ** *p* < 0.05 compared to the “tumor” group. Differences between groups were assessed by one-way ANOVA and in case of significance, a Tukey post-hoc test was used. MMSE—Mini–Mental State Examination, other abbreviations as in Table 1.

## Data Availability

All data generated or analyzed during this study are included in this published article. Primary datasets generated during and/or analyzed during the current study are available from the corresponding author on reasonable request.

## References

[1] Kanner A.M. (2017). Psychiatric comorbidities in new onset epilepsy: Should they be always investigated?. Seizure.

[2] Salpekar J.A., Mula M. (2019). Common psychiatric comorbidities in epilepsy: How big of a problem is it?. Epilepsy Behav..

[3] Vinti V., Dell’Isola G.B., Tascini G., Mencaroni E., Di Cara G., Striano P., Verrotti A. (2021). Temporal Lobe Epilepsy and Psychiatric Comorbidity. Front. Neurol..

[4] Coppola G., Operto F.F., Matricardi S., Verrotti A. (2019). Monitoring and Managing Depression in Adolescents with Epilepsy: Current Perspectives. Neuropsychiatr. Dis. Treat..

[5] Mula M., Kaufman K.R. (2020). Double stigma in mental health: Epilepsy and mental illness. BJPsych Open.

[6] Ribot R., Kanner A.M. (2019). Neurobiologic properties of mood disorders may have an impact on epilepsy: Should this motivate neurologists to screen for this psychiatric comorbidity in these patients?. Epilepsy Behav..

[7] Gulyaeva N.V. (2021). Stress-Associated Molecular and Cellular Hippocampal Mechanisms Common for Epilepsy and Comorbid Depressive Disorders. Biochemistry.

[8] Joëls M. (2001). Corticosteroid Actions in the Hippocampus. J. Neuroendocr..

[9] Joëls M., Karst H., Sarabdjitsingh R.A. (2018). The stressed brain of humans and rodents. Acta Physiol..

[10] Gulyaeva N.V. (2019). Functional Neurochemistry of the Ventral and Dorsal Hippocampus: Stress, Depression, Dementia and Remote Hippocampal Damage. Neurochem. Res..

[11] Gulyaeva N.V. (2019). Biochemical Mechanisms and Translational Relevance of Hippocampal Vulnerability to Distant Focal Brain Injury: The Price of Stress Response. Biochemistry.

[12] Wulsin A.C., Solomon M.B., Privitera M.D., Danzer S.C., Herman J.P. (2016). Hypothalamic-pituitary-adrenocortical axis dysfunction in epilepsy. Physiol. Behav..

[13] Van Campen J.S., Valentijn F.A., Jansen F.E., Joëls M., Braun K.P. (2015). Seizure occurrence and the circadian rhythm of cortisol: A systematic review. Epilepsy Behav..

[14] Vezzani A., Balosso S., Ravizza T. (2019). Neuroinflammatory pathways as treatment targets and biomarkers in epilepsy. Nat. Rev. Neurol..

[15] Koyama R., Ikegaya Y. (2005). To BDNF or Not to BDNF: That Is the Epileptic Hippocampus. Neuroscientist.

[16] Troubat R., Barone P., Leman S., DeSmidt T., Cressant A., Atanasova B., Brizard B., El Hage W., Surget A., Belzung C. (2021). Neuroinflammation and depression: A review. Eur. J. Neurosci..

[17] Brunoni A.R., Lopes M., Fregni F. (2008). A systematic review and meta-analysis of clinical studies on major depression and BDNF levels: Implications for the role of neuroplasticity in depression. Int. J. Neuropsychopharmacol..

[18] Mikulska J., Juszczyk G., Gawrońska-Grzywacz M., Herbet M. (2021). HPA Axis in the Pathomechanism of Depression and Schizophrenia: New Therapeutic Strategies Based on Its Participation. Brain Sci..

[19] Murri M.B., Pariante C., Mondelli V., Masotti M., Atti A.R., Mellacqua Z., Antonioli M., Ghio L., Menchetti M., Zanetidou S. (2014). HPA axis and aging in depression: Systematic review and meta-analysis. Psychoneuroendocrinology.

[20] Thomson F., Craighead M. (2008). Innovative Approaches for the Treatment of Depression: Targeting the HPA Axis. Neurochem. Res..

[21] Kennis M., Gerritsen L., Van Dalen M., Williams A., Cuijpers P., Bockting C. (2020). Prospective biomarkers of major depressive disorder: A systematic review and meta-analysis. Mol. Psychiatry.

[22] Dziurkowska E., Wesolowski M. (2021). Cortisol as a Biomarker of Mental Disorder Severity. J. Clin. Med..

[23] Fischer S., Macare C., Cleare A.J. (2017). Hypothalamic-pituitary-adrenal (HPA) axis functioning as predictor of antidepressant response–Meta-analysis. Neurosci. Biobehav. Rev..

[24] Cano-López I., Gonzalez-Bono E. (2019). Cortisol levels and seizures in adults with epilepsy: A systematic review. Neurosci. Biobehav. Rev..

[25] de Kloet E., Meijer O., de Nicola A., de Rijk R., Joëls M. (2018). Importance of the brain corticosteroid receptor balance in metaplasticity, cognitive performance and neuro-inflammation. Front. Neuroendocr..

[26] Brandner S., Schroeter S., Çalışkan G., Salar S., Kobow K., Coras R., Blümcke I., Hamer H., Schwarz M., Buchfelder M. (2022). Glucocorticoid modulation of synaptic plasticity in the human temporal cortex of epilepsy patients: Does chronic stress contribute to memory impairment?. Epilepsia.

[27] Cano-López I., Hidalgo V., Hampel K.G., Garcés M., Salvador A., González-Bono E., Villanueva V. (2019). Cortisol and trait anxiety as relevant factors involved in memory performance in people with drug-resistant epilepsy. Epilepsy Behav..

[28] de Kloet E.R., Karst H., Joëls M. (2008). Corticosteroid hormones in the central stress response: Quick-and-slow. Front. Neuroendocr..

[29] Maggio N., Segal M. (2010). Corticosteroid Regulation of Synaptic Plasticity in the Hippocampus. Sci. World J..

[30] Joëls M. (2006). Corticosteroid effects in the brain: U-shape it. Trends Pharmacol. Sci..

[31] Basu T., Maguire J., Salpekar J.A. (2021). Hypothalamic-pituitary-adrenal axis targets for the treatment of epilepsy. Neurosci. Lett..

[32] Salpekar J.A., Salpekar J.A., Basu T., Basu T., Thangaraj S., Thangaraj S., Maguire J., Maguire J. (2020). The intersections of stress, anxiety and epilepsy. Int. Rev. Neurobiol..

[33] Bauer M.E., Teixeira A.L. (2021). Neuroinflammation in Mood Disorders: Role of Regulatory Immune Cells. Neuroimmunomodulation.

[34] Nobis A., Zalewski D., Waszkiewicz N. (2020). Peripheral Markers of Depression. J. Clin. Med..

[35] Pfau M.L., Ménard C., Russo S.J. (2018). Inflammatory Mediators in Mood Disorders: Therapeutic Opportunities. Annu. Rev. Pharmacol. Toxicol..

[36] Debnath M., Berk M., Maes M. (2021). Translational evidence for the Inflammatory Response System (IRS)/Compensatory Immune Response System (CIRS) and neuroprogression theory of major depression. Prog. Neuro-Psychopharmacol. Biol. Psychiatry.

[37] Osimo E.F., Pillinger T., Rodriguez I.M., Khandaker G.M., Pariante C.M., Howes O.D. (2020). Inflammatory markers in depression: A meta-analysis of mean differences and variability in 5,166 patients and 5,083 controls. Brain, Behav. Immun..

[38] Enache D., Pariante C.M., Mondelli V. (2019). Markers of central inflammation in major depressive disorder: A systematic review and meta-analysis of studies examining cerebrospinal fluid, positron emission tomography and post-mortem brain tissue. Brain, Behav. Immun..

[39] Jenkins B.J., Hughes S.T.O., Figueras A.C., Jones S.A. (2021). Unravelling the broader complexity of IL-6 involvement in health and disease. Cytokine.

[40] Messaoud A., Rym M., Wahiba D., Neffati F., Najjar M.F., Gobbi G., Manchia M., Valtorta F., Lotfi G., Comai S. (2021). Investigation of the relationship among cortisol, pro-inflammatory cytokines, and the degradation of tryptophan into kynurenine in patients with major depression and suicidal behavior. Curr. Top. Med. Chem..

[41] Ishikawa Y., Furuyashiki T. (2021). The impact of stress on immune systems and its relevance to mental illness. Neurosci. Res..

[42] Vezzani A., Auvin S., Ravizza T., Aronica E., Noebels J.L., Avoli M., Rogawski M.A., Olsen R.V., Delgado-Escueta A.V. (2012). Glia-neuronal interactions in ictogenesis and epileptogenesis: Role of inflammatory mediators. Jasper’s Basic Mechanisms of the Epilepsies.

[43] Vezzani A., Balosso S., Ravizza T. (2012). Inflammation and epilepsy. Handbook of Clinical Neurology.

[44] Barnes S.E., Zera K.A., Ivison G.T., Buckwalter M.S., Engleman E.G. (2021). Brain profiling in murine colitis and human epilepsy reveals neutrophils and TNFα as mediators of neuronal hyperexcitability. J. Neuroinflammation.

[45] Bosco D.B., Tian D., Wu L. (2020). Neuroimmune interaction in seizures and epilepsy: Focusing on monocyte infiltration. FEBS J..

[46] Yamanaka G., Morichi S., Takamatsu T., Watanabe Y., Suzuki S., Ishida Y., Oana S., Yamazaki T., Takata F., Kawashima H. (2021). Links between Immune Cells from the Periphery and the Brain in the Pathogenesis of Epilepsy: A Narrative Review. Int. J. Mol. Sci..

[47] Tan T.H., Perucca P., O’Brien T.J., Kwan P., Monif M. (2021). Inflammation, ictogenesis, and epileptogenesis: An exploration through human disease. Epilepsia.

[48] Terrone G., Salamone A., Vezzani A. (2017). Inflammation and Epilepsy: Preclinical Findings and Potential Clinical Translation. Curr. Pharm. Des..

[49] Xu D., Miller S.D., Koh S. (2013). Immune mechanisms in epileptogenesis. Front. Cell. Neurosci..

[50] Khaboushan A.S., Yazdanpanah N., Rezaei N. (2022). Neuroinflammation and Proinflammatory Cytokines in Epileptogenesis. Mol. Neurobiol..

[51] de Vries E.E., Munckhof B.V.D., Braun K.P., van Royen-Kerkhof A., de Jager W., Jansen F.E. (2016). Inflammatory mediators in human epilepsy: A systematic review and meta-analysis. Neurosci. Biobehav. Rev..

[52] Arulsamy A., Shaikh M.F. (2020). Tumor Necrosis Factor-α, the Pathological Key to Post-Traumatic Epilepsy: A Comprehensive Systematic Review. ACS Chem. Neurosci..

[53] Güneş M., Büyükgöl H. (2020). Relationship between generalized epileptic seizure and neutrophil/lymphocyte ratio, platelet/lymphocyte ratio, and neutrophil mediated inflammation. Int. J. Neurosci..

[54] Alvim M.K.M., Morita-Sherman M.E., Yasuda C.L., Rocha N.P., Vieira L., Pimentel-Silva L.R., Nogueira M.H., Barbosa R., Watanabe N., Coan A.C. (2021). Inflammatory and neurotrophic factor plasma levels are related to epilepsy independently of etiology. Epilepsia.

[55] Shpak A., Guekht A., Druzhkova T., Rider F., Gudkova A., Gulyaeva N. (2021). Increased ciliary neurotrophic factor in blood serum and lacrimal fluid as a potential biomarkers of focal epilepsy. Neurol. Sci..

[56] Iughetti L., Lucaccioni L., Fugetto F., Predieri B., Berardi A., Ferrari F. (2018). Brain-derived neurotrophic factor and epilepsy: A systematic review. Neuropeptides.

[57] Wang X., Hu Z., Zhong K. (2021). The Role of Brain-Derived Neurotrophic Factor in Epileptogenesis: An Update. Front. Pharmacol..

[58] Nowroozi A., Salehi M.A., Mohammadi S. (2021). Brain-derived neurotrophic factor in patients with epilepsy: A systematic review and meta-analysis. Epilepsy Res..

[59] Castrén E., Monteggia L.M. (2021). Brain-Derived Neurotrophic Factor Signaling in Depression and Antidepressant Action. Biol. Psychiatry.

[60] Miyanishi H., Nitta A. (2021). A Role of BDNF in the Depression Pathogenesis and a Potential Target as Antidepressant: The Modulator of Stress Sensitivity “Shati/Nat8l-BDNF System” in the Dorsal Striatum. Pharmaceuticals.

[61] Porter G.A., O’Connor J.C. (2022). Brain-derived neurotrophic factor and inflammation in depression: Pathogenic partners in crime?. World J. Psychiatry.

[62] Chen S., Zhang Y., Yuan Y. (2021). The Combination of Serum BDNF, Cortisol and IFN-Gamma Can Assist the Diagnosis of Major Depressive Disorder. Neuropsychiatr. Dis. Treat..

[63] Galvão A.C.D.M., Almeida R.N., Júnior G.M.D.S., Leocadio-Miguel M.A., Palhano-Fontes F., de Araujo D.B., Lobão-Soares B., Maia-De-Oliveira J.P., Nunes E.A., Hallak J.E.C. (2021). Potential biomarkers of major depression diagnosis and chronicity. PLoS ONE.

[64] Shan D., Zheng Y., Froud K. (2021). Brain-Derived Neurotrophic Factor as a Clinical Biomarker in Predicting the Development of Post-Stroke Depression: A Review of Evidence. Cureus.

[65] Peruga I., Hartwig S., Merkler D., Thöne J., Hovemann B., Juckel G., Gold R., Linker R.A. (2012). Endogenous ciliary neurotrophic factor modulates anxiety and depressive-like behavior. Behav. Brain Res..

[66] Jia C., Brown R.W., Malone H.M., Burgess K.C., Gill W.D., Keasey M.P., Hagg T. (2019). Ciliary neurotrophic factor is a key sex-specific regulator of depressive-like behavior in mice. Psychoneuroendocrinology.

[67] Druzhkova T., Pochigaeva K., Yakovlev A., Kazimirova E., Grishkina M., Chepelev A., Guekht A., Gulyaeva N. (2018). Acute stress response to a cognitive task in patients with major depressive disorder: Potential metabolic and proinflammatory biomarkers. Metab. Brain Dis..

[68] Jones S.A., Jenkins B.J. (2018). Recent insights into targeting the IL-6 cytokine family in inflammatory diseases and cancer. Nat. Rev. Immunol..

[69] Fisher R.S., Cross J.H., French J.A., Higurashi N., Hirsch E., Jansen F.E., Lagae L., Moshé S.L., Peltola J., Roulet Perez E. (2017). Operational classification of seizure types by the International League Against Epilepsy: Position Paper of the ILAE Commission for Classification and Terminology. Epilepsia.

[70] Scheffer I.E., Berkovic S., Capovilla G., Connolly M.B., French J., Guilhoto L., Hirsch E., Jain S., Mathern G.W., Moshe S. (2017). ILAE classification of the epilepsies: Position paper of the ILAE Commission for Classification and Terminology. Epilepsia.

[71] Folstein M.F., Folstein S.E., McHugh P.R. (1975). “Mini-Mental State”. A Practical Method for Grading the Cognitive State of Patients for the Clinician. J. Psychiatr. Res..

[72] Beck A.T., Steer R.A., Brown G.K. (1996). Manual for The Beck Depression Inventory.

[73] Lang U.E., Borgwardt S. (2013). Molecular Mechanisms of Depression: Perspectives on New Treatment Strategies. Cell. Physiol. Biochem..

